# In vitro Activity of Cefiderocol and Comparators against Carbapenem-Resistant Gram-Negative Pathogens from France and Belgium

**DOI:** 10.3390/antibiotics11101352

**Published:** 2022-10-04

**Authors:** Saoussen Oueslati, Pierre Bogaerts, Laurent Dortet, Sandrine Bernabeu, Hend Ben Lakhal, Christopher Longshaw, Youri Glupczynski, Thierry Naas

**Affiliations:** 1 Team ReSIST, LabEx LERMIT, INSERM U1184, School of Medicine, Université Paris-Saclay, 94270 Le Kremlin-Bicêtre, France; 2 Bacteriology-Hygiene Unit, Assistance Publique/Hôpitaux de Paris, Bicêtre Hospital, 94270 Le Kremlin-Bicêtre, France; 3 French National Reference Center for Antibiotic Resistance: Carbapenemase-Producing Enterobacteriaceae, 94270 Le Kremlin-Bicêtre, France; 4 Laboratory of Clinical Microbiology, National Reference Center for Monitoring Antimicrobial Resistance in Gram-Negative Bacteria, CHU UCL Namur, 5530 Yvoir, Belgium; 5 Centre Hospitalier d’Orsay, Service de Réanimation, 91400 Orsay, France; 6 Medical Affairs Europe, Shionogi Europe, London WC2B 6UF, UK

**Keywords:** CFDC, MIC, multidrug-resistant bacteria

## Abstract

Infections with carbapenem-resistant (CR) Gram-negative (GN) pathogens have increased in many countries worldwide, leaving only few therapeutic options. Cefiderocol (CFDC) is approved in Europe for the treatment of aerobic GN infections in adults with limited treatment options. This study evaluated the in vitro activity of cefiderocol and comparators against multidrug-resistant (MDR) bacteria including meropenem-resistant (MR) or pandrug-resistant (PR) GN clinical isolates from France and Belgium. The minimum inhibitory concentrations (MICs) of CFDC were determined by broth microdilution, using iron-depleted cation-adjusted Mueller–Hinton broth, and were compared to those of 10 last-line antibiotics. The MICs were interpreted according to EUCAST and CLSI breakpoints, and in the absence of species-specific breakpoints, non-species-related pharmacokinetic/pharmacodynamic breakpoints were used. Among the 476 isolates tested, 322 were carbapenemase producers (CP), 58 non-CP-CRs, 52 intrinsically CR, 41 expanded-spectrum cephalosporin resistant and 5 were multi-susceptible. Susceptibility to CFDC was high using EUCAST breakpoints 81%, 99% and 84%, and was even higher using CLSI breakpoints to 93%, 100% and 88% for Enterobacterales, *Pseudomonas aeruginosa* and *Acinetobacter baumannii*, respectively. Susceptibility to cefiderocol using non-species-related breakpoints for *Stenotrophomonas maltophilia*, *Achromobacter xylosoxydans* and *Burkholderia cepacia*, was 100%, 100% and 92.3%, respectively. The susceptibility rates were lower with the NDM producers, with values of 48% and 30% using EUCAST breakpoints and 81% and 50% using CLSI breakpoints for Enterobacterales and *Acinetobacter* spp, respectively. CFDC demonstrated high in vitro susceptibility rates against a wide range of MDR GN pathogens, including MR and PR isolates.

## 1. Introduction

Among the antimicrobial agents that belong to the class of beta-lactams, carbapenems display the broadest spectrum of antimicrobial activities and are considered last-resort agents to treat infections caused by extended-spectrum β-lactamase (ESBL)-producing Enterobacterales and multidrug-resistant (MDR) Gram-negative bacilli (GNB) [1,2,3,4]. However, their activity is challenged by the emergence and dissemination of carbapenem-resistant Enterobacterales (CRE) and non-fermenters such as *Pseudomonas aeruginosa* and *Acinetobacter baumannii* [5,6]. Antibiotic resistance among GNB poses a substantial global threat to patients and healthcare systems, often leading to an increased duration of hospital stays, higher medical costs and increased rates of mortality [1,2,3,4]. 

Carbapenem resistance may be the result of combined mechanisms of both outer-membrane permeability defects (e.g., porin defects) and non-carbapenemase β-lactamases (e.g., acquired or overexpressed chromosome-encoded cephalosporinase, and ESBLs) and carbapenemase production [7]. In France, there has been a steady increase in the spread of carbapenemase-producing Enterobacterales (CPE) in recent years [8,9]. The carbapenemases reported among CPE include KPC, NDM, VIM and OXA-48, and although KPC is prevalent in other European countries, OXA-48 remains the most common carbapenemase in France [8,9,10]. Furthermore, a 2018 report from the French National Reference Center (F-NRC) for CPEs showed a notable increase in isolates producing MBLs such as NDM and VIM, as well as the ongoing diversification of OXA-48-type carbapenemases, especially OXA-181 and OXA-244 variants [9,11,12]. These findings, along with the first isolation of IMP-producing Enterobacterales, highlight the evolving and challenging epidemiology of carbapenemases among Enterobacterales in France, and at a larger level in Europe [9,10].

Although the prevalence of infections caused by non-fermenting GN pathogens such as *A. baumannii*, *P. aeruginosa* and *Stenotrophomonas maltophilia* has remained relatively low in France and Belgium, rates of carbapenem resistance among these pathogens are considerably higher than those reported for Enterobacterales [11,13]. Among the 954 *P. aeruginosa* isolates submitted to the F-NRC of antibiotic resistances in 2018, 16.2% produced an ESBL (PER-1, SHV-2a, GES, VEB, OXA), 15.1% were carbapenemase producers (VIM, IMP, DIM, GES) and 2.8% of isolates produced both [11]. Similarly, among the 379 isolates of *A. baumannii*, 96.6% expressed at least one carbapenemase (primarily OXA-23, OXA-72 and NDM-1) together with an ESBL in 2.1% of the isolates [11]. The high levels of resistance among non-fermenters, particularly to carbapenems, reduce the arsenal of effective therapeutics, often making treatment more problematic [2,13,14].

Therapeutic options for carbapenem-resistant (CR) GNB infections in general are limited and many CR pathogens exhibit MDR phenotypes, including all β-lactams (e.g., cephalosporins and penicillins), and other common drug classes such as aminoglycosides and fluoroquinolones [13,14,15]. Furthermore, there has been a consistent rise in the annual number of extensively drug-resistant (XDR) CPEs identified in France since 2012 [8,9,11]. These emerging pathogens are resistant even to last-resort antibiotics such as colistin, or to newly released antibiotics, and are a source of great concern for the treatment of patients [16,17].

Cefiderocol (CFDC) is a novel siderophore cephalosporin developed for the treatment of infections caused by GNB, including those resistant to carbapenems [18]. CFDC is approved in the USA for the treatment of complicated urinary tract infections (cUTIs), including pyelonephritis, hospital-acquired pneumonia and ventilator-acquired pneumonia caused by susceptible Gram-negative microorganisms, and has recently been approved in Europe for the treatment of infections caused by aerobic GNB in adults with limited or no alternative treatment options [18,19]. The structure of CFDC is based around a cephalosporin backbone with the addition of a catechol moiety at the three-position side chain [20,21]. The cephalosporin core enables CFDC to act like other cephalosporins, binding primarily to penicillin-binding proteins and killing bacterial cells by inhibiting peptidoglycan cell wall biosynthesis, and the catechol moiety chelates ferric (Fe-III) iron, mimicking natural siderophores, allowing CFDC to exploit the bacteria’s own active receptor-mediated iron transport system to cross the outer membrane [20,21]. The resulting increase in the periplasmic concentration circumvents non-specific resistance due to porin loss or efflux and enhances CFDC’s activity relative to carbapenems, other cephalosporins and β-lactam/β-lactamase inhibitor combinations [22]. CFDC is active against CR-GNB, including those with derepressed AmpC and/or ESBLs plus porin/efflux pump resistance mechanisms as well as those harboring carbapenemases from different Ambler classes, including KPC, VIM, IMP, NDM and OXA carbapenemases [22,23,24,25,26,27,28,29]. Activity has also been demonstrated against meropenem-resistant and MDR *P. aeruginosa* and *A. baumannii* [28,29,30]. 

Here, we report the antimicrobial activity of CFDC and comparators (aztreonam, amikacin, cefepime, ceftazidime, ceftazidime–avibactam, ceftolozane–tazobactam, ciprofloxacin, meropenem, colistin and tigecycline) against a panel of 476 mostly MDR GNB collected from hospitals in France and Belgium between 2012 and 2019, thus before any clinical use of CFDC (Table 1).

## 2. Results

### 2.1. Activity of Cefiderocol

The in vitro activity of CFDC and comparators was assessed in 476 Gram-negative isolates collected from two National Reference Centers (NRC) for AMR located in France and Belgium (Table 1). The 222 (46.6%) Enterobacterales isolates were from the French NRC for CREs and the remaining 254 (53.4%) bacteria came equally from the two NRCs. These isolates were MDR and of reduced susceptibility/resistant to carbapenems.

Susceptibility to CFDC was high for all the tested MDR GNB (Table 2). Susceptibility to CFDC using EUCAST breakpoints (<2 mg/L) was 81%, 99% and 84%, which rose with the investigational CLSI breakpoints (<4 mg/L) to 93%, 100% and 88% for Enterobacterales, *P. aeruginosa* and *A. baumannii*, respectively (Table 2) [31,32]. Susceptibility to CFDC using EUCAST non-species-related breakpoints for *S. maltophilia*, *A. xylosoxydans* and *B. cepacia* were 100%, 100% and 92.3%, respectively. The susceptibility rates were lower with NDM producers, with values of 48% and 30% using EUCAST breakpoints [31] and 81% and 50% using CLSI breakpoints [32,33] for Enterobacterales and *A. baumannii*, respectively.

The MIC_50_ and MIC_90_ values for CFDC and comparators are reported by pathogen in Table 3 and Table 4. 

Among all Enterobacterales, the susceptibility rates to CFDC (81%) were comparable to those for colistin (84%) and tigecycline (73%); on the other hand, a higher proportion of Enterobacterales isolates were susceptible to CFDC than to ceftazidime/avibactam (63%) and ceftolozane/tazobactam (19%). Among the non-fermenting GNB, 84% of the A. baumannii isolates were susceptible to CFDC, which was higher than all other comparators apart from colistin (88%), and 99% of P. aeruginosa isolates were susceptible to CFDC, which was higher than all other comparators also including colistin (97%).

### 2.2. Cefiderocol Activity among Enterobacterales Isolates

The MIC_50_ of CFDC was at 1 mg/L, while those of other drugs were >64 mg/L for ceftazidime, 64 mg/L for ceftolozane–tazobactam, >32 mg/L for aztreonam, >16 mg/L for cefepime, 8 mg/L for meropenem and amikacin, >4 mg/L for ciprofloxacin, 4 mg/L for ceftazidime–avibactam, ≤0.25 mg/L for tigecycline and ≤0.5 mg/L for colistin.

The MIC_90_ for the Enterobacterales of CFDC was 4 mg/L (Table 3), while those of the comparator antibiotics were >64 mg/L for ceftolozane–tazobactam, meropenem, ceftazidime, ceftazidime–avibactam and amikacin; >32 mg/L for aztreonam; >16 mg/L for cefepime; >8 mg/L for colistin; >4 mg/L for ciprofloxacin; and 2 mg/L for tigecycline. 

Among the 80 meropenem-susceptible isolates (MIC ≤ 2 mg/L), 10 (12.5%) were resistant to CFDC and 8 (10%) were resistant to ceftazidime–avibactam. Among the 44 meropenem intermediate isolates (considered susceptible with increased dosing regimen (MIC of 4 and 8 mg/L)), 6 (13.6%) were resistant to CFDC and 17 (38.6%) were resistant to ceftazidime–avibactam. Finally, with the 98 meropenem-resistant isolates (MIC > 8 mg/L), 27 (27.5%) were resistant to CFDC while 58 (59.2%) were resistant to ceftazidime–avibactam. Among the latter, 37 (64%) were still susceptible to CFDC. Among the 27 CFDC-resistant isolates, 18 were NDM producers.

Non-CP-producing Enterobacterales isolates with a reduced susceptibility to carbapenems had a susceptibility rate of 85% to CFDC, which was globally comparable to the susceptibility rates observed for ceftazidime–avibactam (90%), amikacin (81%) and colistin (79%). 

A total of 92% of the KPC producers were susceptible to CFDC, with an MIC_50/90_ of 1/4 mg/L, results that are similar to those of ceftazidime/avibactam (94% susceptibility, and an MIC_50/90_ of 2/8). The only other competitive comparators were amikacin (76%), tigecycline (76%) (0.5/1) and colistin (62%) (0.5/>8). For all the other antibiotics, the MIC_50/90_ values were superior or equal to the upper limit of the concentration range used in the MIC testing.

OXA-48-like producing Enterobacterales were susceptible to a larger number of antibiotics compared with the KPC producers (Table 3). A total of 90% of the OXA-48-like producers were susceptible to CFDC, with low MIC_50/90_ values of 1 and 2 mg/L, respectively. Its direct competitors were ceftazidime–avibactam (96%; 1/8), meropenem (78% S+I; 4/32), amikacin (90%; ≤4/8), colistin (94%; ≤0.5/1) and tigecycline (75%; ≤0.5/2).

In total, 69% of the Enterobacterial isolates producing NDM, VIM or IMP carbapenemases (Table 3) were susceptible to CFDC with an MIC_50/90_ of 2/8 mg/L. The only comparator antibiotics with high susceptibility rates were colistin (91%; ≤0.5/1) and tigecycline (75%; ≤0.25/2).

### 2.3. Cefiderocol Activity against Meropenem-Resistant Non-Fermenters

Carbapenemase-producing *P. aeruginosa* were susceptible only to CFDC (0.25/1) and to colistin (1/2) (Table 3). The same resistance trend was observed for carbapenemase-producing *A. baumannii* strains (CFDC (1/8) and colistin (1/4)), except that the latter were also susceptible to tigecycline (1/2) (Table 3). The only unexpected result was the overall low activity of ceftolozane–tazobactam against those non-CP *P. aeruginosa* isolates (48.4% of susceptibility).

Among the 120 *P. aeruginosa* isolates tested, only one isolate exhibited an MIC value of CFDC of 4 mg/L, which is considered resistant by EUCAST, but still susceptible by CLSI. Noteworthy, in *P. aeruginosa,* CFDC was active against all MBL producers, while all comparators were below 20% except for colistin (97%). Thirteen *A. baumannii* (15.9%) had MICs > 2 mg/L, among which seven were NDM producers, three were ESBL producers (two PER and one VEB) and three were OXA-23 producers. 

### 2.4. Cefiderocol Activity against Intrinsically Meropenem-Resistant Non-Fermenters 

Among the intrinsically meropenem-resistant non-fermenters (*S. maltophilia*, *A. xylosoxidans*, *Elizabethkingia* spp.) or frequently meropenem-resistant non-fermenters (*B. cepacia*), 51 of the 52 isolates (98%) were susceptible to CFDC (Table 4).

Overall, a higher proportion of meropenem-resistant non-fermenters were susceptible to CFDC than to any of the other comparator antimicrobials tested (Table 4). Minocyclin for *S. maltophilia* (100% both), ceftazidime–avibactam for *B. cepacia* (92.3% vs. 69.2%) and colistin for *A. xylosoxidans* (100% vs. 33.3%) were the best comparators.

## 3. Discussion

In the French SIDERO-WT study, CFDC displayed excellent in vitro activity, but since only very few meropenem-resistant Enterobacterales were included, it was not possible to assess CFDC activity versus other antimicrobials as comparators for these isolates [29]. In the present evaluation of 476 GNB isolates, of which 472 were MDR, from France and Belgium (of which 66% were meropenem R (MIC > 8 mg/L)), CFDC demonstrated substantial in vitro activity. These isolates were from hospitals in France and Belgium and were mainly isolated in 2018 and 2019, thus before any clinical use of CFDC. Notably, CFDC demonstrated substantial activity against all isolates of *P. aeruginosa*, most *A. baumannii* and intrinsically meropenem-resistant GN non-fermenters such as *S. maltophilia* and *A. xylosoxidans*, where all other comparators demonstrated much lower susceptibility rates. Overall, more isolates were susceptible to CFDC than to a key subset of other currently available antimicrobial agents including the β-lactam/β-lactamase inhibitor combinations ceftazidime–avibactam and ceftolozane–tazobactam, and colistin, which is often considered a last resort molecule for MDR GNB infections. 

Carbapenem resistance among GNB in France is steadily rising and poses a substantial threat to patients and healthcare systems, often leading to greater rates of mortality, morbidity and increased burden on hospitals [8,9,11]. In France and Belgium, OXA-48 remains the most common carbapenemase reported nationally among CPEs [8,9,11]. The OXA-48 variants, OXA-181 and the difficult-to-detect OXA-244 are increasingly isolated among CPEs, including ESBL producers. There is now increasing concern over the emergence of OXA-48-mediated resistance to new antibiotic regimens such as ceftazidime–avibactam [34]. Therefore, there is a continued need for new antibiotics and antibiotic regimens with activity against OXA-48-like producers, among others. Despite the dominance of OXA-48 in France and Belgium, over recent years there has been a notable shift in resistance mechanisms, with an increase in MBL producers such as NDM and VIM [8,9,11] and the first isolation of IMP-producing Enterobacterales. This shift to MBL-mediated resistance is of concern as new β-lactam/β-lactamase inhibitor combination therapies, including ceftazidime–avibactam, ceftolozane–tazobactam and meropenem–vaborbactam, are known to lack efficacy against MBLs. As such, these agents cannot be proposed for empirical treatment against infections that are suspected to involve MBL-producing GNB [35,36]. Previous reports from the SIDERO-WT study have shown potent in vitro activity of CFDC against carbapenemase-producing isolates including MBL producers [27,29]. In these studies, only a few MBL producers were included. Here, in our study, 54 MBLs, among which were 21 NDM producers, were studied. Only 50% of the NDM-producing Enterobacterales were susceptible, which was yet much higher than the comparators, except for colistin and tigecycline. In general, MICs for cefiderocol with Enterobacterales-producing MBLs are close to the breakpoints with 28% (*n* = 15) with an MIC = 1 mg/L, 20% (*n* = 11) with an MIC = 2 mg/L, and 20% (*n* = 11) with an MIC = 4 mg/L. A possible explanation for these higher MICs as compared to other carbapenemases [37] is likely due to the fact that MBLs and especially NDM have stronger hydrolytic activity against expanded-spectrum cephalosporins, including CFDC, as suggested by the addition the MBL inhibitor, dipicolinic acid, that reduced the MICs of CFDC against previously non-susceptible Enterobacterales isolates [38]. Based on resistance reports, the increased copy number of NDM may increase CFDC MIC values in the absence of CirA mutations, which is the iron transporter involved in CFDC uptake. However, when NDM overexpression is associated with mutations of the *cirA* gene, a loss of fitness was observed in these isolates. Of note, the combination of mutations in the iron transport genes and the expression of the NDM enzyme was found in CREs in China, way before CFDC was used in clinical practice [39]; thus, it has been suggested that resistance to CFDC may be the consequence of previous antibiotic treatments, to cancer therapies or to so far unknown mechanisms of selection [40,41,42].

Furthermore, in instances where resistance is not due to carbapenemase production, CFDC has demonstrated in vitro activity against isolates with AmpC, ESBLs, porin mutations and efflux pump upregulation.

Although the prevalence of infections caused by non-fermenting GNB currently remains relatively low in France, there is growing concern regarding the high propensity of these isolates to develop resistance, and the resulting depletion of available effective treatment options [13,43]. In this study, the CFDC activity exceeded that of all the tested comparators except colistin against meropenem-resistant isolates of *S. maltophilia*, *P. aeruginosa* and *A. baumannii*.

These findings are in line with previous reports in other countries, which demonstrated the potent in vitro activity of CFDC against MDR Enterobacterales, MDR *A. baumannii*, MDR *P. aeruginosa* and *S. maltophilia* [37,44]. Additionally, novel agents such as ceftazidime/avibactam, imipenem/relebactam and meropenem/vaborbactam have recently been approved against antibiotic-resistant GNB as they are effective against Enterobacterales-producing KPC but have limited or no efficacy against CR *A. baumannii* [35,43]. 

Overall, there are very few antimicrobial agents available to clinicians to treat patients infected with CR GNB, and the agents that are available are often associated with considerable toxicities and increasing resistance. Colistin is effective against a wide range of CR-GNB, and in this study, colistin was the only agent with comparable activity to CFDC against non-fermenters collected from patients with nosocomial pneumonia or bloodstream infections (BSI). However, the usage of colistin is associated with a potential risk of nephro- and neurotoxicity [45] and several species of Enterobacterales have demonstrated intrinsic colistin resistance. Additionally, in this study, fewer meropenem-resistant *S. maltophilia* isolates were susceptible to colistin than to CFDC. The in vivo results confirmed the excellent behavior of CFDC for the treatment of MDR GNB in bloodstream infections [46,47,48,49,50,51]. CFDC has also shown to be a promising new treatment option for patients with bone and joint infections due to CR *A. baumannii* and appears to be well tolerated for prolonged durations [49,50].

## 4. Materials and Methods

### 4.1. Bacteria

The tested isolates (Table 1) were from the French and Belgium National Reference Centers for antibiotic resistances among GNB and comprised (i) 222 isolates of Enterobacterales, selected to represent diverse carbapenemase producers and isolates with carbapenem resistance via combinations of porin loss with AmpC or ESBL activity; (ii) 120 isolates of *P. aeruginosa*, selected to represent producers of MBLs and GES carbapenemases, along with isolates that produced ESBLs and were carbapenem-resistant via porine OprD loss; (iii) 82 MDR isolates of *A. baumannii* expressing various carbapenemases, including NDM and/or various OXA carbapenemases; and (iv) 52 GNB naturally resistant to carbapenems: 25 *S. maltophilia*, 13 *B. cepacia*, 12 *A. xylosoxidans* and 2 *Elizabethkingia* sp.

These isolates were selected by both NRCs to represent the French and Belgium epidemiology of carbapenem-resistant GNB and challenging isolates expressing rare carbapenemases. As CFDC had not previously been tested, these isolates were chosen based on their carbapenem/expanded spectrum susceptibility profiles and their enzymatic content. Almost all the isolates tested were submitted for an investigation of MDR/XDR resistance phenotypes by hospital laboratories in France and Belgium between 2012 and 2019, thus before any clinical use of CFDC. Carbapenemases and ESBL enzymes were identified by PCR of their encoding genes or by whole-genome sequencing (WGS). Carbapenem resistance due to porin loss combined with ESBL or AmpC activity was inferred from previous susceptibility results and the absence of carbapenemase, as confirmed by PCR or WGS. Species identification was by matrix-assisted laser desorption ionization-time of flight (MALDI-TOF) mass spectroscopy.

### 4.2. Antimicrobial Susceptibility Testing

The MICs were determined using frozen 96-well broth microdilution panels with a pre-loaded antibiotic growth medium supplied by International Health Management Associates, Inc. (IHMA; Schaumburg, IL, USA). CFDC was tested in iron-depleted cation-adjusted Mueller–Hinton broth (ID-CAMHB), as recently approved by the CLSI ([32]; http://clsi.org/standards/micro/microbiology-files/, accessed on 1 September 2022), whereas the comparators were tested in cation-adjusted Mueller–Hinton broth (CAMHB). The strains were grown overnight on a non-selective agar media. Two to three colonies were resuspended in 3 mL sterile 0.85% NaCl in order to obtain a 0.5 McFarland suspension. One milliliter of this suspension was further diluted in 29 mL of sterile water, of which 10 μL were then added to each well, and the plates were subsequently incubated for 16–20 h at 35 °C, as recommended by the manufacturer and EUCAST guidelines [31]. Quality control testing was performed on each day of testing using *E. coli* ATCC 25922, *K. pneumoniae* ATCC 700603 and *P. aeruginosa* ATCC 27853 to ensure the stability of the panels and the validity of the test methods. The comparator antibiotics were for Enterobacterales, *Pseudomonas* spp., *Acinetobacter* spp., *B. cepacia*, *A. xylosoxydans* and *Elizabethkingia* spp. meropenem, ceftazidime, ceftazidime–avibactam (4 μg/mL), cefepime, ceftolozane–tazobactam (4 μg/mL), aztreonam, colistin, amikacin, ciprofloxacin and tigecycline, all sourced by IHMA. For *S. maltophilia*, cotrimoxazole, levofloxacin and minocycline were tested instead of cefepime, aztreonam and ciprofloxacin.

The MIC results of CFDC were interpreted using EUCAST breakpoint [31] values of S ≤ 2 mg/L and R >2   mg/L for Enterobacterales, *P. aeruginosa*, *Acinetobacter* spp and *S. maltophilia,* and for the other tested bacteria, non-species-related PK/PD values (≤2 mg/L ) were used; the Investigational CLSI MIC breakpoints for the same bacteria were used with values of S ≤ 4  mg/L and R ≥ 16  mg/L, which correspond to those when CFDC was in trial. The MICs of the comparator antibiotics were interpreted using EUCAST guidelines where available, the exceptions being ceftazidime and cefepime for *Acinetobacter* spp., for which only the CLSI breakpoints are available [32,33].

### 4.3. Ethics

Ethics approval was not required as all the bacterial isolates were from the French or Belgium NRC for antibiotic resistances and thus were anonymized and unrelated to the patients.

## 5. Conclusions

The increasing incidence and diversification of carbapenem resistance among GNB is of growing concern in France and in Belgium, as a shift toward more difficult-to-treat pathogens is putting pressure on the already limited available treatment options. CFDC demonstrates substantial and broad in vitro activity against a wide range of MDR pathogens, and even XDR GNB. The findings from this study are in line with those from previous reports and suggest that CFDC may offer an invaluable treatment option in the fight against antimicrobial-resistant GNB, particularly for carbapenem-resistant non-fermenters and MBL producers, especially *Acinetobacter baumannii*, for which there are currently few approved effective therapies. Colistin was the only other agent with similar activity as CFDC against meropenem-resistant GNB. It should be emphasized that CFDC displays much more favorable pharmacokinetic parameters (tissue diffusion and use in renal impairment) than colistin and tigecycline, which will be an important factor for choosing an adequate therapy for infections due to multidrug infections.

In addition to aztreonam, CFDC is the other beta-lactam with activity against MBL-producing CREs. Our results, along with other in vitro and surveillance studies, showed that CFDC MIC values are higher against NDM-producing isolates than VIM-producing isolates. Nevertheless, clinical studies demonstrated that NDM-producing CRE infections with CFDC MICs of 4 µg/mL, which corresponds to the CLSI susceptibility breakpoint, could be successfully treated [48]. The recent IDSA guidance and ESCMID guidelines provide recommendations on when and how to use the new antimicrobial agents, especially to prevent irrational use and the emergence of resistance [52,53]. Neither of them recommends a second agent to be used with the new antibiotics for the treatment of CRE infections. Even though resistance for each of the new agents has been described, great susceptibility rates are described globally, with some regional variations. Overall susceptibility rates are reduced for ceftazidime–avibactam, meropenem–vaborbactam and imipenem–relebactam in regions where MBLs are prevalent, and CFDC MICs are higher where NDM-producing CREs are more prevalent. This underlines the need for rapid diagnostic tests for resistance mechanisms that will improve the surveillance and diagnosis of CRE and, hence, the selection of the most appropriate antibiotic agent [54,55].

## Figures and Tables

**Table 1:** Panel of tested isolates.

ß-lactam Resistant Mechanism	Carbapenemase-Producers																												Impermeability							ESC Resistant ^1^									WT	IR^2^	Total
Genus/Species	NDM	VIM	IMP	GIM	AIM	SPM	DIM	SIM	LMB	TMB	KPC	GES	IMI	SME	FRI	OXA- 48 like	OXA-372	OXA-198	OXA- 23	OXA- 24/40	hyper OXA- 51	OXA-58	OXA-143	OXA-48/NDM	OXA-48/VIM	NDM/VIM	NDM/KPC	NDM/OXA-23	ESBL + Dporin	AMPC + Dporin	ESBL/AmpC/Dporin	Efflux	OprD	AmpC/Efflux	AmpC/Efflux/OprD	Hyper K1	pAmpC	CTX-M	VEB	BEL	PER	SCO/RTG/Carb	PME	ESBL-OXA			
*E. coli*	10	3	3								4					21								4		1			1	1							2	6	1		2						59
*Klebsiella*	8	10	7								12	1				21								7					12							2	1	3			1			1	2		88
*Enterobacter*		2	2	1						1	5	1	4		1	3											1			18	3								1		1			1			45
*Serratia*			1								2			3		3														1														1			11
*Citrobacter*		2							1		1					2	1							2	2						1																12
*Morganella*																														1																	1
*Providencia*	2																																														2
*Salmonella enterica*	1																																														1
*Proteus*																																					1										1
*Hafnia alvei*																														2																	2
*P. aeruginosa*	4	52	9	2	1	1	1				4	8						3														3	1	2	12				5	2	1		1	1			113
*P. putida*		2	1																																												3
*P. stutzeri*		1	1				1																																								3
*P. fluorescens*		1																																													1
*A. xylosoxydans*		1																																												11	12
*A. baumannii*	9	2	3					1				8							19	9	9	8	2					3											2		1	3		1	2		82
*B. cepacia*																																														13	13
*S. maltophilia*																																														25	25
*E. miricola*																																														1	1
*E.meningoseptica*																																														1	1
Total	34	76	27	3	1	1	2	1	1	1	28	18	4	3	1	50	1	3	19	9	9	8	2	13	2	1	1	3	13	23	4	3	1	2	12	2	4	9	9	2	6	3	1	5	4	51	476

^1^ ESC: Expanded-spectrum cephalosporin resistant; ^2^ IR = Intrinsic carbapenem-resistance

**Table 2:** MIC distributions of cefiderocol by resistance mechanism and species group.

Mechanism	Total # of Isolates	# of Isolates per MIC (mg/L)													% Susceptible Isolates at Breakpoints of (mg/L)	
		≤0.03	0.06	0.125	0.25	0.5	1	2	4	8	16	32	64	>64	≤2 ^1^	≤4 ^2^
**Enterobacterales**	222	6	5	13	18	31	67	39	27	5	5	1	2	3	81	93
Non CPE	67	2	1	2	10	10	17	15	8	0	0	1	1	0	85	97
KPC	24	0	0	1	0	5	11	5	1	1	0	0	0	0	92	96
other class A GES, IMI, SME, fri…	9	0	0	2	1	2	2	0	1	1	0	0	0	0	78	89
MBLs	54	1	1	3	3	3	15	11	11	1	3	1	0	1	69	89
NDM	21	0	0	0	0	2	4	4	7	1	2	0	0	1	48	81
VIM	17	0	0	0	1	0	6	6	2	0	1	1	0	0	76	88
IMP	13	1	1	3	2	0	4	0	2	0	0	0	0	0	85	100
other MBLs (LMB, GIM, TMB)	3	0	0	0	0	1	1	1	0	0	0	0	0	0	100	100
OXA-48	51	3	2	5	4	11	16	5	3	2	0	0	0	0	90	96
Multi-Carbas	17	0	1	0	0	0	6	3	3	0	1	0	1	2	59	76
*P. aeruginosa*	120	2	10	22	29	30	17	9	1	0	0	0	0	0	99	100
Non-CP, ESBLs	31	0	1	7	8	7	4	3	1	0	0	0	0	0	97	100
MBLS	77	2	8	13	19	18	12	5	0	0	0	0	0	0	100	100
VIM	56	1	7	12	14	11	8	3	0	0	0	0	0	0	100	100
IMP	11	0	0	1	4	5	1	0	0	0	0	0	0	0	100	100
NDM, GIM, DIM, SPM, AIM	10	1	1	0	1	2	3	2	0	0	0	0	0	0	100	100
OXA-198, GES, KPC	12	0	1	2	2	5	1	1	0	0	0	0	0	0	100	100
*A. baumannii*	82	1	7	11	6	15	16	13	3	3	1	0	0	6	84	88
ESBL, Non CP	26	0	0	4	1	6	5	7	0	0	1	0	0	2	88	88
OXA-23, 40, 58, 143	40	1	7	7	5	5	10	2	1	1	0	0	0	1	93	95
NDM-like	10	0	0	0	0	0	0	3	2	2	0	0	0	3	30	50
VIM, IMP	6	0	0	0	0	4	1	1	0	0	0	0	0	0	100	100
*S. maltophilia*	25	22	2	1	0	0	0	0	0	0	0	0	0	0	100	100
*B. cepacia*	13	10	0	1	0	1	0	0	0	1	0	0	0	0	92.3	92.3
*A. xylosoxidans*	12	0	0	1	4	4	2	1	0	0	0	0	0	0	100	100
*Elizabethkingia sp*	2	0	0	1	0	0	1	0	0	0	0	0	0	0	100	100

^1^ EUCAST susceptibility breakpoints 202; ^2^ CLSI susceptibility breakpoints 2022.

**Table 3:** In vitro activity of cefiderocol and comparators against Enterobacterales, *P. aeruginosa* and *A. baumannii* with reduced susceptibility to carbapenems received at the French and Belgium NRC for antibiotic resistance in GN according to EUCAST guidelines.

Species	Resistance Mechanism (# of Isolates)	Antimicrobial Agent	MIC (mg/L)				S/I/R		
			Range	MIC_50_	MIC_90_		S (%)	I (%) ^1^	R (%)
***Enterobacterales***									
	Total (222)	Cefiderocol	≤0.03–>64	1	4		**81**	/	19
		Ceftolozane–tazobactam	≤0.03–>64	64	>64		19	/	81
		Cefepime	≤0.5–>16	>16	>16		14	10	76
		Ceftazidime	0.12–>64	>64	>64		9	8	83
		Ceftazidime–avibactam	0.06–>64	4	>64		63	/	37
		Aztreonam	≤0.5–>32	>32	>32		14	4	82
		Meropenem	0.06–>64	8	>64		36	20	44
		Amikacin	≤4–>64	≤4	>64		70	/	30
		Ciprofloxacin	≤0.25–>4	>4	>4		30	5	65
		Colistin	≤0.5–>8	≤0.5	>8		84	/	16
		Tigecycline	≤0.25–>4	≤0.25	2		73	/	27
	Non-CPE (67)	Cefiderocol	0.12–64	1	4		**85**	/	15
		Ceftolozane–tazobactam	0.12–>64	16	>64		30	/	70
		Cefepime	≤0.5–>16	16	>16		16	16	68
		Ceftazidime	0.25–>64	>64	>64		7	9	84
		Ceftazidime–avibactam	0.06–>64	2	12		90	/	10
		Aztreonam	≤0.5–>32	>32	>32		10	4	85
		Meropenem	0.06–>64	0.5	32		73	6	21
		Amikacin	≤4–>64	≤4	32		81	9	19
		Ciprofloxacin	≤0.25–>4	1	>4		34	6	60
		Colistin	≤0.5–>8	≤0.5	>8		79	/	21
		Tigecycline	≤0.25–>4	0.5	2		69	/	31
	Class A, KPC producers (24)	Cefiderocol	0.12–8	1	4		**92**	/	8
		Ceftolozane–tazobactam	0.25–>64	32	>64		29	/	71
		Cefepime	2–>16	16	>16		26	9	65
		Ceftazidime	0.25–>64	64	>64		21	3	76
		Ceftazidime–avibactam	0.12–>64	2	8		94	/	6
		Aztreonam	2–>32	>32	>32		6	9	85
		Meropenem	0.25–>64	32	>64		15	20	65
		Amikacin	≤4–>64	4	32		76	/	24
		Ciprofloxacin	≤0.25–>4	1	>4		41	6	53
		Colistin	≤0.5–>8	1	>8		62	/	38
		Tigecycline	≤0.25–>4	0.5	1		76	/	24
	Class A, other carbapenemase (IMI, NMC-A, SME, GES, FRI-1) producers (10)	Cefiderocol	0.12–8	0.5	4		**78**	/	22
		Ceftolozane–tazobactam	0.25–32	0.5	32		78	/	22
		Cefepime	≤0.5–>16	≤0.5	16		78	11	11
		Ceftazidime	0.25–>64	0.5	>64		78	0	22
		Ceftazidime–avibactam	0.25–4	0.5	8		100	/	0
		Aztreonam	1–>32	4	>32		11	33	56
		Meropenem	8–>64	64	>64		0	11	89
		Amikacin	≤4–8	≤4	8		100	/	0
		Ciprofloxacin	≤0.25–>4	≤0.25	>4		89	0	11
		Colistin	1–>8	>8	>8		11	/	89
		Tigecycline	≤0.25–2	≤0.25	2		67	/	33
	Class B, MBLs total (54)	Cefiderocol	0.03–>64	2	8		**69**	/	31
		Ceftolozane–tazobactam	64–>64	>64	>64		0	/	100
		Cefepime	2–>16	>16	>16		0	2	98
		Ceftazidime	64–>64	>64	>64		0	0	100
		Ceftazidime–avibactam	32–>64	>64	>64		0	/	100
		Aztreonam	≤0.5–>32	>32	>32		20	0	80
		Meropenem	0.5–>64	32	>64		7	24	69
		Amikacin	≤4–>64	16	>64		46	/	54
		Ciprofloxacin	0.25–>4	>4	>4		22	6	72
		Colistin	≤0.5–>8	≤0.5	1		91	/	9
		Tigecycline	≤0.25–>4	≤0.25	2		70	/	30
	Class B, NDM producers (21)	Cefiderocol	0.5–>64	2	16		**48**	/	52
		Ceftolozane–tazobactam	>64	>64	>64		0	/	100
		Cefepime	16–>16	>16	>16		0	/	100
		Ceftazidime	>64	>64	>64		0	0	100
		Ceftazidime–avibactam	>64	>64	>64		0	/	100
		Aztreonam	≤0.5–>32	>32	>32		14	0	86
		Meropenem	16–>64	32	>64		0	0	100
		Amikacin	≤4–>64	16	>64		33	0	67
		Ciprofloxacin	2–>4	>4	>4		0	0	100
		Colistin	≤0.5–>8	≤0.5	1		48	/	52
		Tigecycline	≤0.25–4	≤0.25	2		52	/	48
	Class B, VIM producers (17)	Cefiderocol	0.25–>64	2	16		**76**	/	24
		Ceftolozane–tazobactam	>64	>64	>64		0	/	100
		Cefepime	16–>16	>16	>16		0	0	100
		Ceftazidime	>64	>64	>64		0	0	100
		Ceftazidime–avibactam	32–>64	>64	>64		0	/	100
		Aztreonam	≤0.5–>32	>32	>32		18	0	82
		Meropenem	1–>64	32	>64		6	29	65
		Amikacin	≤4–>32	16	32		24	/	76
		Ciprofloxacin	0.25–>4	>4	>4		24	0	76
		Colistin	≤0.5–>8	≤0.5	1		94	/	6
		Tigecycline	≤0.25–>4	0.5	1		76	/	24
	Class B, IMP producers (13)	Cefiderocol	0.03–4	0.25	4		**85**	/	15
		Ceftolozane–tazobactam	64–>64	>64	>64		0	/	100
		Cefepime	2–>16	8	>16		0	0	100
		Ceftazidime	64–>64	>64	>64		0	0	100
		Ceftazidime–avibactam	32–>64	>64	>64		0	/	100
		Aztreonam	≤0.5–>32	16	>32		38	0	62
		Meropenem	1–64	4	32		15	62	23
		Amikacin	≤4–16	≤4	16		85	/	15
		Ciprofloxacin	≤0.25–>4	≤0.25	>4		46	23	31
		Colistin	≤0.5–>8	≤0.5	1		92	/	8
		Tigecycline	≤0.25–>4	≤0.25	1		92	/	8
	Other class B producers (GIM, LMB, TMB) (3)	Cefiderocol	0.5–2	1	2		**100**	/	0
		Ceftolozane–tazobactam	64–>64	>64	>64		0	/	100
		Cefepime	2–8	8	8		33	0	67
		Ceftazidime	>64	>64	>64		0	0	100
		Ceftazidime–avibactam	32–>64	>64	>64		0	/	100
		Aztreonam	0.5–32	>32	>32		0	0	100
		Meropenem	0.5–32	32	>64		33	0	67
		Amikacin	4	4	4		100	/	0
		Ciprofloxacin	≤0.25–>4	≤0.25	>4		67	0	33
		Colistin	≤0.5–>8	≤0.5	1		100	/	0
		Tigecycline	≤0.25–>1	≤0.25	1		67	/	33
	Class D, OXA-48 producers (50) + OXA-372 (1)	Cefiderocol	≤0.03–8	1	2		**90**	/	10
		Ceftolozane–tazobactam	≤0.03–>64	8	>64		37	/	73
		Cefepime	1–>16	16	>16		25	14	61
		Ceftazidime	0.12–>64	>64	>64		15	20	65
		Ceftazidime–avibactam	0.12–>64	1	8		94	/	6
		Aztreonam	1–>32	>32	>32		24	4	73
		Meropenem	0.06–>64	4	32		45	33	22
		Amikacin	≤4–>64	4	8		90	/	10
		Ciprofloxacin	≤0.25–>4	>4	>4		35	4	61
		Colistin	≤0.5–>8	1	2		94	/	6
		Tigecycline	≤0.25–>4	≤0.25	2		75	/	25
	Multiple Carbapenemase producers (17)	Cefiderocol	0.06–>64	2	>64		**76**	/	24
		Ceftolozane–tazobactam	>64	>64	>64		0	/	100
		Cefepime	4–>64	>64	>64		0	6	94
		Ceftazidime	32–>64	>64	>64		0		100
		Ceftazidime–avibactam	16–>64	>64	>64		0	/	100
		Aztreonam	2–>16	>16	>16		0		100
		Meropenem	≤4–>32	>32	>32		18		82
		Amikacin	≤0.5–1	≤0.5	1		29	/	71
		Ciprofloxacin	≤4–>64	>64	>64		0		100
		Colistin	4–>8	>4	>4		94	/	6
		Tigecycline	≤0.25–4	≤0.25	4		76	/	24
*Acinetobacter* spp.									
	Total (*n* = 82)	Cefiderocol	0.03–>64	1	8		**84**	/	16
		Ceftolozane–tazobactam	1–>64	64	>64		13	/	87
		Cefepime	2–>16	>16	>16		0	7	93
		Ceftazidime	8–>64	>64	>64		0	2	98
		Ceftazidime–avibactam	8–>64	>64	>64		5	/	95
		Aztreonam^2^	8–>32	>32	>32		0	5	95
		Meropenem	2–>64	>64	>64		2	11	87
		Amikacin	4–>64	64	>64		28	/	72
		Ciprofloxacin	0.5–>4	>4	>4		0	9	91
		Colistin	0.5–8	1	4		88	/	12
		Tigecycline	0.25–>4	1	2		48	/	52
	Non-CP (*n* = 26)	Cefiderocol	0.12–>64	1	4		**88**	/	12
		Ceftolozane–tazobactam	2–>64	>64	>64		15	/	85
		Cefepime	2–>16	>16	>16		0	12	88
		Ceftazidime	8–>64	>64	>64		0	4	96
		Ceftazidime–avibactam	8–>64	>64	>64		4	/	96
		Aztreonam^2^	>32	>32	>32		0	0	100
		Meropenem	2–>64	32	>64		8	27	65
		Amikacin	≤4–>64	64	>64		23	/	77
		Ciprofloxacin	≤0.25–>4	>4	>4		0	15	85
		Colistin	≤0.5–4	1	2		96	/	4
		Tigecycline	≤0.25–>4	1	2		46	/	54
	Class D, OXA carbapenemase (*n* = 40)	Cefiderocol	0.03–>64	0.25	2		**93**	/	7
		Ceftolozane–tazobactam	2–>64	>64	>64		12	/	88
		Cefepime	8–>16	>16	>16		0	5	95
		Ceftazidime	8–>64	>64	>64		0	3	97
		Ceftazidime–avibactam	8–>64	64	>64		8	/	92
		Aztreonam^2^	8–>32	>32	>32		0	5	95
		Meropenem	8–>64	>64	>64		0	5	95
		Amikacin	4–>64	64	>64		25	/	75
		Ciprofloxacin	4–>4	>4	>4		0	0	100
		Colistin	<0.5–4	1	4		85	/	15
		Tigecycline	0.25–>4	1	2		42	/	57
	Class B, NDM (*n* = 10)	Cefiderocol	2–>64	4	>64		**30**	/	70
		Ceftolozane–tazobactam	>64	>64	>64		0	/	100
		Cefepime	>16	>16	>16		0	0	100
		Ceftazidime	>64	>64	>64		0		100
		Ceftazidime–avibactam	>64	>64	>64		0	/	100
		Aztreonam^2^	>32	>32	>32		0	0	100
		Meropenem	>64	32	>64		0	0	100
		Amikacin	4–>64	4	>64		50	/	50
		Ciprofloxacin	>4	>4	>4		0	0	100
		Colistin	0.5–4	1	2		90	/	10
		Tigecycline	0.25–2	0.5	2		60	/	40
	Class B, other MBLS (*n* = 6)	Cefiderocol	0.5–1	0.5	1		**100**	/	0
		Ceftolozane–tazobactam	1–>64	>64	>64		34	/	66
		Cefepime	8–>16	16	>16		0	16	84
		Ceftazidime	32–>64	>64	>64		0		100
		Ceftazidime–avibactam	32–>64	>64	>64		0	/	100
		Aztreonam^2^	16–>32	32	>32		0	34	66
		Meropenem	16–>64	64	>64		0	0	100
		Amikacin	≤4–>64	64	>64		34	/	66
		Ciprofloxacin	0.25–>4	0.25	>4		0	50	50
		Colistin	1–4	2	4		66	/	34
		Tigecycline	0.25–1	0.25	1		16	/	84
*Pseudomonas* spp.									
	Total (*n* = 120)	Cefiderocol	0.03–4	0.25	1		**99**	/	1
		Ceftolozane–tazobactam	0.5–>64	>64	>64		17	/	83
		Cefepime	2–>16	>16	>16		0	15	85
		Ceftazidime	2–>64	64	>64		0	5	95
		Ceftazidime–avibactam	2–>64	32	>64		22	/	78
		Aztreonam	2–>32	32	>32		0	11	89
		Meropenem	1–>64	64	>64		3	13	84
		Amikacin	4–>64	32	>64		34	/	66
		Ciprofloxacin	0.25–>4	>4	>4		0	14	86
		Colistin	0.5–>8	1	2		97	/	3
		Tigecycline^3^	1–>4	>4	>4		0	/	100
	Non-CP-CR (*n* = 31)	Cefiderocol	0.06–4	0.25	2		**97**	/	3
		Ceftolozane–tazobactam	0.5–>64	4	>64		52	/	48
		Cefepime	2–>16	>16	>16		0	23	77
		Ceftazidime	2–>64	>64	>64		0	6	94
		Ceftazidime–avibactam	2–>64	16	>64		48	/	52
		Aztreonam	8–>32	>32	>32		0	0	100
		Meropenem	1–>64	16	>64		6	29	65
		Amikacin	≤4–>64	16	>64		58	/	42
		Ciprofloxacin	≤0.25–>4	>4	>4		0	16	84
		Colistin	≤0.5–>8	1	2		97	/	3
		Tigecycline^3^	2–>4	>4	>4		0	/	100
	OXA-198, GES, KPC (*n* = 12)	Cefiderocol	0.06–2	0.5	1		**100**	/	0
		Ceftolozane–tazobactam	4–>64	16	>64		25	/	75
		Cefepime	2–>16	>16	>16		0	17	83
		Ceftazidime	4–>64	>64	>64		0	17	83
		Ceftazidime–avibactam	2–>64	8	>64		67	/	33
		Aztreonam	8–>32	>32	>32		0	0	100
		Meropenem	16–>64	>64	>64		0	0	100
		Amikacin	≤4–>64	64	>64		42	/	58
		Ciprofloxacin	≤0.25–>4	>4	>4		0	8	92
		Colistin	≤0.5–2	1	2		100	/	0
		Tigecycline^3^	2–>4	>4	>4		0	/	100
	Class B, MBL (*n* = 77)	Cefiderocol	0.03–4	0.25	1		**100**	/	0
		Ceftolozane–tazobactam	0.5–>64	>64	>64		3	/	97
		Cefepime	2–>16	>16	>16		0	12	88
		Ceftazidime	2–>64	64	>64		0	3	97
		Ceftazidime–avibactam	2–>64	64	>64		5	/	95
		Aztreonam	2–>32	16	>32		0	17	83
		Meropenem	1–>64	64	>64		1	8	91
		Amikacin	≤4–>64	32	>64		23	/	77
		Ciprofloxacin	≤0.25–>4	>4	>4		0	14	86
		Colistin	≤0.5–>8	1	2		97	/	3
		Tigecycline^3^	1–>4	>4	>4		0	/	100
	VIM (*n* = 56)	Cefiderocol	≤0.06–2	0.25	1		**100**	/	0
		Ceftolozane–tazobactam	2–>64	>64	>64		2	/	98
		Cefepime	8–>16	>16	>16		0	13	88
		Ceftazidime	4–>64	64	>64		0	2	98
		Ceftazidime–avibactam	4–>64	64	>64		5	/	95
		Aztreonam	2–>32	8	>32		0	20	80
		Meropenem	4–>64	64	>64		0	7	93
		Amikacin	8–>64	64	>64		16	/	84
		Ciprofloxacin	≤0.25–>4	>4	>4		0	16	84
		Colistin	≤0.5–4	1	2		98	/	2
		Tigecycline^3^	1–>4	>4	>4		0	/	100
	IMP (*n* = 11)	Cefiderocol	0.12–1	0.25	2		**100**	/	0
		Ceftolozane–tazobactam	64–>64	4	>64		0	/	100
		Cefepime	>16	>16	>16		0	0	100
		Ceftazidime	>64	>64	>64		0	0	100
		Ceftazidime–avibactam	>64	16	>64		0	/	100
		Aztreonam	2–>32	>32	>32		0	18	82
		Meropenem	8–>64	16	>64		0	9	91
		Amikacin	≤4–>64	16	>64		36	/	64
		Ciprofloxacin	≤0.25–>4	>4	>4		0	9	91
		Colistin	≤0.5–>4	1	2		91	/	9
		Tigecycline^3^	1–>4	4	>4		0	/	100
	Other MBLs (NDM, GIM, DIM, SPM, AIM) (*n* = 10)	Cefiderocol	≤0.06–2	0.5	2		**100**	/	0
		Ceftolozane–tazobactam	0.5–>64	>64	>64		10	/	90
		Cefepime	2–>16	>16	>16		0	20	80
		Ceftazidime	4–>64	>64	>64		0	10	90
		Ceftazidime–avibactam	2–>64	>64	>64		10	/	90
		Aztreonam	8–>32	>32	>32		0	0	100
		Meropenem	2–>64	16	>64		10	10	80
		Amikacin	≤4–>64	16	>64		50	/	50
		Ciprofloxacin	≤0.25–>4	>4	>4		0	10	90
		Colistin	≤0.5–2	1	2		100	/	0
		Tigecycline^3^	2–>4	>4	>4		0	/	100

^1^ “I” refers to susceptible with high exposure according to EUCAST guidelines. ^2^ *A. baumannii* are intrinsically resistant to aztreonam. ^3^ *P. aeruginosa* are intrinsically resistant to tigecycline.

**Table 4:** In vitro activity of cefiderocol and comparators against Gram-negative pathogens isolated from hospitals in France and Belgium according to EUCAST breakpoints.

Species	# of Isolates	Antimicrobial Agent	MIC (mg/L)				S/I/R		
			Range	MIC_50_	MIC_90_		S (%)	I (%)	R (%)
*S. maltophilia*	(*n* = 25)	Cefiderocol	≤0.03–0.12	≤0.03	0.06		**100**	/	0
		Ceftolozane–tazobactam	≤0.03–>64	32	>64		24	/	76
		Ceftazidime	0.5–>64	64	>64		0	16	84
		Ceftazidime–avibactam	0.12–>64	64	>64		20	/	80
		Meropenem	2–>64	>64	>64		4	0	96
		SXT	≤0.25–>16	0.5	>16		0	72	0
		Amikacin	≤4–>64	>64	>64		16	/	84
		Levofloxacin	≤1–8	≤1	8		0	60	40
		Colistin	≤0.5–>8	4	>8		32	/	32
		Minocycline	≤2	≤2	≤2		100	0	0
		Tigecycline	≤0.25–2	≤0.25	1		76	/	24
*B. cepacia*	(*n* = 13)	Cefiderocol	≤0.03–8	≤0.03	0.5		**92.3**	/	7.7
		Ceftolozane–tazobactam	1–>64	4	>64		61.5	/	38.5
		Cefepime	8–>16	>16	>16		0	15.4	84.6
		Ceftazidime	4–>64	8	>64		0	61.5	38.5
		Ceftazidime–avibactam	4–64	4	32		69.2	/	30.8
		Aztreonam	>32	>32	>32		0	0	100
		Meropenem	16–64	32	64		0	0	100
		Amikacin	64–>64	>64	>64		0	/	100
		Ciprofloxacin	0.5–>4	1	>4		0	7.7	92.3
		Colistin ^1^	>8	>8	>8		0	/	100
		Tigecycline	1–>4	4	>4		0	/	100
*A. xylosoxidans*	(*n* = 12)	Cefiderocol	0.25–2	0.5	1		**100**	/	0
		Ceftolozane–tazobactam	16–>64	>64	>64		0	/	100
		Cefepime	>16	>16	>16		0	0	100
		Ceftazidime	16–>64	>64	>64		0	0	100
		Ceftazidime–avibactam	8–64	64	>64		16.6	/	83.3
		Aztreonam	>32	>32	>32		0	0	100
		Meropenem	0.5–32	32	32		16.7	16.7	66.7
		Amikacin	64–>64	>64	>64		0	/	100
		Ciprofloxacin	2–>8	2	>4		0	0	100
		Colistin	0.5–>8	4	>8		33.3	/	66.7
		Tigecycline	0.25–4	1	2		25	0	75
*Elizabethkingia* sp.	(*n* = 2)	Cefiderocol	0.12–1						
		Ceftolozane–tazobactam	16–32						
		Cefepime	8–32						
		Ceftazidime	16–>64						
		Ceftazidime–avibactam	>64						
		Aztreonam	>32						
		Meropenem	32						
		Amikacin	4>64						
		Ciprofloxacin	0.25–0.5						
		Colistin	16						
		Tigecycline	0.5–4						

^1^*B. cepacia* isolates are naturally resistant to colistin.

## Data Availability

Not applicable.
